# Chromatographic Examinations of Tea's Protection Against Lipid Oxidative Modifications

**DOI:** 10.1080/15376510701624050

**Published:** 2008-06-23

**Authors:** Wojciech Łuczaj, Tomasz Welerowicz, Elżbieta Skrzydlewska, Bogusław Buszewski

**Affiliations:** Department of Analytical Chemistry, Medical University of Białystok, Poland; Department of Environmental Chemistry and Ecoanalytics, Nicolaus Copernicus University, Toruń, Poland; Department of Analytical Chemistry, Medical University of Białystok, Poland; Department of Environmental Chemistry and Ecoanalytics, Nicolaus Copernicus University, Toruń, Poland

**Keywords:** Antioxidants, Black Tea, Ethanol, Lipid Peroxidation, Rats

## Abstract

Ethanol metabolism is accompanied by generation of free radicals that damage cell components, especially lipids. The present study was designed to investigate the efficacy of the preventive effect of black tea on the lipid oxidative modifications in different tissues (plasma, liver, brain, kidney, stomach, lung, intestine, and spleen) of 12-month-old rats chronically intoxicated with ethanol. Ethanol intoxication caused changes in the level/activity of antioxidants that led to the significant increase in the level of lipid oxidative modification products. Oxidative modifications were estimated by measuring lipid hydroperoxides, malondialdehyde, and 4-hydroxynonenal by high-performance liquid chromatography (HPLC) and by spectrophotometric determination of conjugated dienes. These lipid-modification marker levels were increased in almost all examined tissues (3%–71%) after ethanol intoxication. Described changes were in accordance with the liver level of the most often used marker of arachidonic acid oxidation, isoprostane (8-isoPGF_2α_), determined by the LC/MS system. Administration of black tea to ethanol-intoxicated rats remarkably prevents the significant increase (by about 15%–42%) in concentrations of all measured parameters regarding all examined tissues, but especially the plasma, liver, brain, stomach, and spleen. The preventive effect of black tea in the other organs (kidney, lung, intestine) caused a decrease in examined markers in a smaller degree (by about 7%–28%). To determine in the liver the major constituents of black tea mainly responsible for antioxidative action such as catechins and theaflavins, which were absorbed in organism, the present study indicates their protective effect against ethanol-induced oxidative modifications of lipids.

## INTRODUCTION

Physiological conditions of the organism are characterized by redox balance. However, several xenobiotics are increasingly recognized as toxic substances that disturb cellular metabolism including redox balance. In most cases this is connected with xenobiotics metabolism. One such compound is ethanol that is oxidated into acetaldehyde, which is oxidated to acetate mainly in the liver, and these processes are accompanied by free radical formation. It has been shown that chronic ethanol intoxication enhances generation mainly of superoxide radical and hydrogen peroxide (Kukielka and Cederbaum 1994). An increase in superoxide radical generation is caused by an increase in the level of NADH produced during ethanol as well as its metabolite acetaldehyde oxidation (Sochmann 1994). A decrease in the NAD/NADH ratio causes conversion of xanthine dehydrogenase into xanthine oxidase, the cytosolic enzyme responsible for superoxide radical generation (Kato et al. 1990). The increase in NADH concentration is also responsible for enhanced release from ferritin iron (II) ions that catalyze free radical reactions (Kukielka and Cederbaum 1992). Chronic alcohol intoxication is also accompanied by changes in activity of ethanol metabolizing enzymes and in consequence by an increase in acetaldehyde accumulation (Lukivskaya and Buko 1993). In such a situation xanthine oxidase may also catalyze superoxide radical generation using acetaldehyde as a substrate (Halliwell and Gutteridge 2001). Moreover, increased generation of oxygen and ethanol-derived free radicals has been observed at the microsomal level, especially through the intervention of the ethanol-inducible cytochrome P450 isoform (Poli 1993). An increase in the free radical level during ethanol intoxication, which is accompanied by changes in antioxidative status during ethanol intoxication, leads to oxidative stress formation (Kurose et al. 1996). Oxidative stress results in enhanced lipid peroxidation and changes in structure and function of other important cellular components, such as protein and DNA (Wang et al. 1990; Rouach et al. 1997).

Therefore, the efficient antioxidants especially natural ones are looked for. One of such potentially health-promoting beverages drunk by most of people is tea. It was generally believed that only green tea prepared by dehydration of *Camellia sinensis* leaves, which contain monomeric polyphenols, possesses antioxidant properties (Graham 1992). However recent investigations indicate that black tea obtained by fermentation of tea leaves and containing only a small amount of monomeric polyphenols–catechins, and big amount of multimeric polyphenols–theaflavins and thearubigins whose biological activities are less documented but still extensively examined, also reveals antioxidant abilities (Frei and Higdon 2003; Łuczaj and Skrzydlewska 2004; Łuczaj et al. 2006; Graham 1992). As a result, black tea has been proved to protect against cancer progression and heart diseases (Dufresne and Farnworth 2001; Yang et al. 2000; Rietveld and Wiseman 2003). However, the biological activity of black tea as a source of antioxidants requires further investigation.

The present study was designed to investigate the influence of black tea on the lipid peroxidation process in different tissues of rats chronically intoxicated with ethanol.

## MATERIAL AND METHODS

### Black Tea

Black tea–*Camellia sinensis* (*Linnaeus*) *O. Kuntze* (standard research blends–lyophilized extract)–was provided by TJ Lipton (Englewood Cliffs, NJ) and was dissolved in drinking water at a concentration of 3 g/L. Tea was prepared three times per week and stored at 4°C until use. The content of drinking vessels was renewed every day. Black tea extract contained catechins (epigallocatechin gallate [EGCG]: 4.84 mg/g dried extract; epigallocatechin [EGC]: 0.74 mg/g dried extract; epicatechin [EC]: 0.94 mg/g dried extract) and theaflavins (theaflavin [TF_1_]; theaflavin 3-gallate [TF_2_A]; theaflavin 3′-gallate [TF_2_B]; theaflavin 3,3′-digallate [TF_3_] in the amount of 156.16 mg/g dried extract for all four TFs). The levels of catechins and TFs were determined by modified high-performance liquid chromatography (HPLC) methods of Mattila et al. (2000) and Lee et al. (2000).

### Animals

Twelve-month-old male Wistar rats were used for the experiment. They were housed in groups with free access to a granular standard diet and water and maintained under a normal light-dark cycle. All experiments were approved by the Local Ethic Committee in Białystok (Poland) referring to Polish Act Protecting Animals of 1997.

The animals were divided into the following groups:

- The control group was treated intragastrically with 1.8 mL of physiological saline every day for 4 weeks (n = 6).
- The black tea group had been given black tea solution ad libitum instead of water for 1 week. Next it was treated intragastrically with 1.8 mL of physiological saline and received black tea solution ad libitum instead of water every day for 4 weeks (n = 6).
- The ethanol group was treated intragastrically with 1.8 mL of ethanol in doses from 2.0 to 6.0 g/kg body weight every day for 4 weeks. The dose of ethanol was gradually increased by 0.5 g/kg body weight every 3 days (n = 6).
- The ethanol and black tea group was given black tea solution ad libitum instead of water for 1 week. Next it was treated intragastrically with 1.8 mL of ethanol in doses from 2.0 to 6.0 g/kg body weight and received black tea solution ad libitum instead of water every day for 4 weeks.

### Preparation of Tissue

After the above procedure, the rats were sacrificed under ether anesthesia (six animals in each group). Blood was taken by cardiac puncture. Livers, brains, kidneys, stomachs, intestines, spleens, and lungs were removed quickly and placed in iced 0.15 M NaCl solution, perfused with the same solution to remove blood cells, blotted on filter paper, weighed, and homogenized in 9 mL ice-cold 0.25 M sucrose and 0.15 M NaCl with the addition of 6 μL 250 mM BHT (butylated hydroxytoluene) in ethanol to prevent the formation of new peroxides during the assay. Homogenization procedure was performed under standardized conditions; 10% homogenates were centrifuged at 10.000 × g for 15 min at 4°C, and the supernatant was kept on ice until assayed.

### Biochemical Assays

The levels of catechins and teaflavins in the liver were determined by the modified high-performance liquid chromatography method of Lee et al. (2000). The method included digestion of tissue samples with β-D-glucuronidase and sulfatase, followed by extraction with ethyl acetate and subsequent separation by HPLC with electrochemical detection. For the analysis of the catechins as well as the theaflavins, binary gradient elution was used. The mobile phase consisted of two phosphate buffers (solution A and solution B) with different amounts of acetonitrile and tetrahydrofuran. Different gradient programs were used for the analysis of catechins and theaflavins. The eluent was monitored by the amperometric detector with potential settings at 390 mV for catechins and 540 mV for theaflavins.

Lipid peroxidation was estimated by measuring conjugated dienes (CDs), lipid hydroxyperoxides (LOOHs), malondialdehyde (MDA), 4-hydoxynoenal (HNE), and 8-isoPGF_2α_ levels. The level of conjugated dienes was measured spectrophotometrically at 234 nm (Recknagel and Glende 1984). The HPLC method with spectrophotometric detector (λ = 292 nm) was used to measure lipid hydroperoxides (Tokumaru et al. 1995). The method involved the oxidation of 1-naphthyldiphenylphosphine into its oxide. The separation was performed on octadecyl column RP C_18_ and 85% methanol was used as an eluent. Malondialdehyde was assayed as malondialdehyde-thiobarbituric acid adducts by HPLC with spectrafluorometric detection (excitation λ = 532 nm, emission λ = 553 nm) (Londero and Greco 1996). The procedure involved a protein precipitation step, acidic hydrolysis, and adducts with thiobarbituric acid (TBA) formation. HPLC separation of MDA-TBA adducts was performed with the mobile phase consisting of 40:60 (v/v) 0.05 M methanol-potassium phosphate buffer, pH 6.8 on RP C_18_ column. 4-Hydroxynonenal (4-HNE) was assayed as a fluorometric derivative with 1,3-cyclohexandione (CHD) separated on RP C_18_ column by HPLC with spectrafluorometric detection (excitation λ = 380 nm, emission λ = 445 nm) (Yoshino et al. 1986). 8-Iso-prostaglandine F_2α_ (8-isoPGF_2α_) was assayed by the emodified LC-MS method of Waugh et al. (1997). The lipid fraction was isolated from the liver homogenate by Folch extraction. To extract of 8-iso-PGF_2α_, the samples were purified by using SEP-PAK C18 column containing octadecylsilyl silica gel (Waters Associates, USA). 8-IsoPGF_2α_ was analyzed by HPLC and detected using electrospray ionization mass spectrometry. The separation was carried out on RP C_18_ with a linear gradient with solvent A (acetic acid adjusted to pH 5.7 with ammonium hydroxide) and solvent B (acetonitrile/methanol, 95/5).

Glutathione peroxidase (EC.1.11.1.6) activity was measured in the liver spectrophotometrically using a technique based on Paglia and Valentine (1967). Using this technique, GSSG formation was assayed by measuring the conversion of nicotinamide adenine dinucleotide phosphate (NADPH) to NADP. One unit of activity was defined as the amount of enzyme catalyzing the conversion of 1 μmol of NADPH/min per mg protein, at 25°C and pH 7.4.

Glutathione (GSH) concentration was measured using the Bioxytech GSH-400 test. The method proceeded in two steps. The first step leads to the formation of substitution products between a patented reagent and all mercaptans (RSH) that are present in the sample. The second step specifically transforms the substitution products obtained with GSH into a chromophoric thione whose maximal absorbance wavelength is 400 nm.

The HPLC method with spectrophotometric detection (λ = 294 nm) was used to determine the level of vitamin E (De Leenher et al. 1979). The vitamin E was extracted from liver homogenate with hexane containing 0.025% butylated hydroxytoluene. The hexane phase was removed and dried with sodium sulfate, and 50 μL of the hexane extract was injected on the RP C_18_ column. The mobile phase consisted of 5/95 (v/v) water-methanol.

### Statistical Analysis

The data obtained in this study are expressed as mean ± SD. The data were analyzed by use of standard statistical analyses, one-way ANOVA with Scheffe's F test for multiple comparisons to determine significance between different groups. The values for *p* < 0.05 were considered significant.

## RESULTS

Responding to the enhancement of ethanol metabolism, liver is the most proper organ for evaluation of lipid peroxidation, whose parameters have been thoroughly described. It has been shown that catechins and theaflavins were not detectable in the liver of rats from control and the ethanol group, while they were detected in the liver of rats drinking black tea. However, the levels of epicatechin, epigallocatechin, and epigallocatechin gallate in the liver of rats from the ethanol and black tea group were lower (decreased by 10%, 8%, and 11%, respectively) than those in the liver of rats in the black tea group (Table 1). Similarly to catechins, the levels of theaflavin, theaflavin 3-gallate, theaflavin 3′-gallate, and theaflavin 3,3′-digallate in the liver of rats in the ethanol and black tea group were lower (decreased by 6%, 5%, 9%, and 8%, respectively) in comparison to black tea group (Table 1).

**TABLE 1:** The levels of catechins and theaflavins in the liver of 12-month-old rats receiving black tea, ethanol, and ethanol and black tea

	Groups			
	
Parameter	Control	Black tea	Ethanol	Ethanol + black tea
*EC* (ng/g tissue)	n.d.	140 ± 9	n.d.	126 ± 10^b^
*EGC* (ng/g tissue)	n.d.	231 ± 19	n.d.	213 ± 14^b^
*EGCG* (ng/g tissue)	n.d.	246 ± 13	n.d.	218 ± 12^b^
*TF_1_* (ng/g tissue)	n.d.	22.4 ± 0.6	n.d.	21.0 ± 1.3^b^
*TF_2_A* (ng/g tissue)	n.d.	57.2 ± 1.7	n.d.	54.0 ± 1.8^b^
*TF_2_B* (ng/g tissue)	n.d.	17.2 ± 0.7	n.d.	15.6 ± 0.8^b^
*TF_3_* (ng/g tissue)	n.d.	20.4 ± 0.2	n.d.	18.8 ± 0.9^b^

a Significantly different from control, *p* < 0.05.

b Significantly different from green tea group, *p* < 0.05.

c Significantly different from ethanol group, *p* < 0.05.

n.d., not detected.

The levels of lipid peroxidation products in the liver are presented in Table 3. It is shown that ethanol intoxication caused an increase in lipid peroxidation products–first (CD by about 19%), intermediate (LOOH by about 68%), and final (MDA by about 33%, HNE by about 34%, 8-isoPGF_2α_ by about 78%). However, drinking of black tea caused a significant decrease in comparison with control group in the level of conjugated dienes (by about 17%), LOOH (by about 3%), and MDA (by about 38%). Therefore, ethanol given to rats drinking black tea caused a significantly smaller increase in the level of LOOH (by about 21%), MDA (by about 32%), HNE (by about 25%), and 8-isoPGF_2α_ (by about 14%). In comparison to the control group, ethanol administration to rats drinking black tea caused an increase in the liver level of LOOH (by about 60%) and 8-isoPGF_2α_ (by about 74%).

Table 2 shows the level/activity of liver antioxidants participating in prevention against lipid peroxidation. It has been proved that the liver activity of glutathione peroxidase was significantly decreased after ethanol intoxication (by about 24%). Drinking of black tea didn't cause changes in the activity of this enzyme. Alcohol administration to rats drinking black tea caused a decrease (by about 8%) in the activity of GSH-Px in comparison with the control group. The liver levels of examined nonenzymatic antioxidants (GSH and vitamin E) were also decreased (by about 26% for GSH and by about 23% for vitamin E) after ethanol administration. However, black tea caused an increase (by about 7%) in the level of GSH. Ethanol given to rats drinking black tea caused a significantly smaller decrease than alcohol ingested alone (by about 25% for GSH and by about 22% for vitamin E).

**TABLE 2:** Antioxidative parameters in the liver of 12-month-old rats receiving black tea, ethanol, and ethanol and black tea

	Groups			
	
Parameter	Control	Black tea	Ethanol	Ethanol + black tea
*GSH-Px* (U/mg protein)	31.8 ± 1.8	32.6 ± 1.7	24.0 ± 1.8^a^,^b^	29.3 ± 1.7^a^,^b^
*GSH* (μmol/g tissue)	0.99 ± 0.04	1.06 ± 0.06^a^	0.73 ± 0.05^a^,^b^	0.97 ± 0.06^a^-^c^
Vitamin E (nmol/g tissue)	24.8 ± 1.6	25.9 ± 1.7	19.1 ± 1.4^a^,^b^	24.4 ± 1.8^c^

a Significantly different from control, *p* <0.05.

b Significantly different from green tea group, *p* <0.05.

c Significantly different from ethanol group, *p* <0.05.

It has been shown that ethanol intoxication enhanced lipid peroxidation also in other tissues of rat such as plasma, brain, lung, stomach, kidney, spleen, and intestine (Table 3). The brain and intestine level of conjugate dienes were significantly increased after ethanol intoxication (by about 3% and 14%, respectively). However, drinking of black tea alone caused a significant decrease in the plasma, brain, and lung level of this parameter (decrease respectively by about 25%, 6%, and 4%), while a small decrease was observed in the stomach, kidney, and spleen. Drinking ethanol and black tea caused an increase in the level of CDs in all examined tissues but only in the lung and spleen the level of conjugate dienes was significantly smaller than in alcohol group (by about 7% and 8%, respectively).

**TABLE 3:** The levels of lipid peroxidation products: conjugate dienes (CDs), lipid hydroxyperoxides (LOOHs), malondialdehyde (MDA), 4-hydroxynonenal (4-HNE), and 8-isoPGF_2α_ in the liver of 12-month-old rats receiving black tea, ethanol, and ethanol and black tea

	Groups			
	
Parameter	Control	Black tea	Ethanol	Ethanol + black tea
CD (μmol/g tissue)	1.08 ± 0.02	0.89 ± 0.01^a^	1.33 ± 0.13^a^,^b^	1.26 ± 0.22^b^
LOOH (μmol/g tissue)	13.60 ± 2.35	13.20 ± 2.77^a^	42.60 ± 0.03^a^,^b^	33.70 ± 0.09^a^-^c^
MDA (nmol/g tissue)	1.12 ± 0.21	0.69 ± 0.20^a^	1.68 ± 0.52^a^,^b^	1.14 ± 0.23^b^,^c^
HNE (nmol/g tissue)	2.25 ± 0.30	1.90 ± 0.25	3.39 ± 0.45^a^,^b^	2.53 ± 0.33^b^,^c^
8-isoPGF_2α_ (ng/g tissue)	2.44 ± 0.31	2.28 ± 0.21	11.06 ± 0.62^a^,^b^	9.52 ± 0.34^a^-^c^

a Significantly different from control, *p* < 0.05.

b Significantly different from green tea group, *p* < 0.05.

c Significantly different from ethanol group, *p* < 0.05.

**TABLE 4:** The levels of lipid peroxidation products (CD, LOOH, MDA, and HNE) in the plasma, brain, lung, stomach, kidney, spleen, and intestine of 12-month-old rats receiving black tea, ethanol, and ethanol and black tea

		Paremeter			
		
Tissue	Groups	CD (μmol/g tissue [mL])	LOOH (μmol/g tissue [mL])	MDA (nmol/g tissue[mL])	HNE (nmol/g tissue[mL])
Plasma	Control	0.20 ± 0.03	13.41 ± 1.70	0.81 ± 0.07	0.30 ± 0.02
	Black tea	0.15 ± 0.02^a^	13.39 ± 2.03	0.74 ± 0.05	0.25 ± 0.03^a^
	Ethanol	0.22 ± 0.02^b^	21.01 ± 3.80^a^,^b^	1.08 ± 0.05^a^,^b^	0.43 ± 0.03^a^,^b^
	Ethanol + black tea	0.21 ± 0.02^b^	15.30 ± 2.30^c^	0.83 ± 0.14^c^	0.36 ± 0.03^a^-^c^
Brain	Control	0.96 ± 0.03	19.00 ± 3.16	0.52 ± 0.12	4.01 ± 0.27
	Black tea	0.90 ± 0.01^a^,^b^	15.98 ± 8.53^b^	0.46 ± 0.11	2.58 ± 0.20^a^
	Ethanol	0.99 ± 0.01^a^	35.80 ± 3.67^a^	0.63 ± 0.19^a^,^b^	7.17 ± 0.56^a^
	Ethanol + black tea	0.97 ± 0.04^b^	21.00 ± 7.4^b^,^c^	0.56 ± 0.12^b^	4.19 ± 0.32^b^,^c^
Lung	Control	1.35 ± 0.03	23.63 ± 0.79	0.8 ± 0.07	0.40 ± 0.06
	Black tea	1.29 ± 0.08^a^	23.50 ± 0.65	0.78 ± 0.09	0.38 ± 0.07
	Ethanol	1.49 ± 0.03^b^	29.54 ± 0.83^b^	0.95 ± 0.06^a^,^b^	0.51 ± 0.07^a^,^b^
	Ethanol + black tea	1.39 ± 0.05^b^,^c^	25.93 ± 0.58^a^-^c^	0.84 ± 0.08	0.44 ± 0.04
Stomach	Control	0.90 ± 0.10	17.48 ± 0.58	0.46 ± 0.05	0.89 ± 0.10
	Black tea	0.87 ± 0.05	16.97 ± 0.67	0.40 ± 0.03	0.87 ± 0.07
	Ethanol	0.99 ± 0.15	24.73 ± 0.49^a^,^b^	0.58 ± 0.06^a^,^b^	1.13 ± 0.11^a^,^b^
	Ethanol + black tea	0.94 ± 0.05^b^	18.09 ± 0.47^b^,^c^	0.49 ± 0.05^b^,^c^	0.94 ± 0.08^c^
Kidney	Control	1.11 ± 0.06	25.90 ± 0.59	1.89 ± 0.10	0.73 ± 0.02
	Black tea	1.10 ± 0.08	23.86 ± 0.79^a^	1.57 ± 0.10^a^	0.72 ± 0.04
	Ethanol	1.14 ± 0.09	33.51 ± 1.03^a^,^b^	2.02 ± 0.20^b^	0.84 ± 0.06^a^,^b^
	Ethanol + black tea	1.13 ± 0.03	27.74 ± 0.52^a^-^c^	1.91 ± 0.08^b^	0.78 ± 0.04^a^^b^
Spleen	Control	1.16 ± 0.13	15.07 ± 0.10	0.90 ± 0.02	0.29 ± 0.05
	Black tea	1.04 ± 0.07	14.21 ± 0.57^a^	0.81 ± 0.03^a^	0.28 ± 0.04
	Ethanol	1.27 ± 0.18^b^	29.17 ± 1.17^a^,^b^	1.24 ± 0.02^a^,^b^	0.36 ± 0.08
	Ethanol + black tea	1.17 ± 0.09^c^	16.82 ± 0.57^a^-^c^	1.12 ± 0.02^a^-^c^	0.31 ± 0.07
Intestine	Control	0.88 ± 0.05	17.88 ± 3.17	0.62 ± 0.06	0.85 ± 0.04
	Black tea	0.80 ± 0.02	16.95 ± 0.64^a^	0.57 ± 0.04	0.83 ± 0.09
	Ethanol	1.02 ± 0.22^a^,^b^	25.59 ± 1.30^b^	0.69 ± 0.09^b^	0.90 ± 0.08
	Ethanol + black tea	0.92 ± 0.14	18.52 ± 0.37^b^,^c^	0.65 ± 0.07^b^	0.89 ± 0.07

a Significantly different from control, *p* <0.05.

b Significantly different from green tea group, *p* <0.05.

c Significantly different from ethanol group, *p* <0.05.

Black tea given alone caused a significant decrease in the level of LOOH in the kidney, intestine, and spleen (by about 8%, 5%, and 6%, respectively). However, administration of alcohol also caused a significant increase in the level of lipid hydroperoxides in plasma, brain, stomach, kidney, and spleen (by about 36%, 47%, 71%, 23%, and 52%, respectively). Ethanol given to rats drinking black tea caused a significantly smaller increase in the LOOH level than alcohol ingested alone in all examined tissues (by about 27% for plasma, 41% for brain, 12% for lung, 27% for stomach, 17% for kidney, 42% for spleen, and 28% for intestine).

The same direction of changes was observed in the malondialdehyde level. A significant increase in the level of this marker was observed in the ethanol group in all tissues except kidney and intestine (by about 25% for plasma, 17% for brain, 16% for lung, 21% for stomach, and 27% for spleen). Black tea caused a significant decrease in MDA level only in the kidney and spleen (by 17% and 10%, respectively). Black tea given with alcohol caused a significant decrease in the MDA level in plasma, stomach, and spleen (by about 23%, 15%, and 21%, respectively) in comparison to the ethanol group.

The least visible changes were observed in the level of the other oxidative marker–4-HNE. However, a significant increase in the 4-HNE level was observed after ethanol intake in all examined tissues except spleen and intestine (by about 30% for plasma, 34% for liver, 44% for brain, 21% for lung and stomach, and 13% for kidney). Besides, drinking black tea caused a significant decrease of the 4-hydroksynonenal level in the plasma and brain (by 17% and 36%, respectively) in comparison with the control group. Moreover, in comparison to the alcohol group, black tea administrated with alcohol also caused a significant decrease in the level of 4-HNE in plasma, brain, and stomach (by about 16%, 41%, and 17%, respectively).

It should be stressed that in most cases the levels of all above parameters of lipid peroxidation in tissues of rats drinking alcohol and black tea were similar to the control group.

## DISCUSSION

It was revealed that, during ethanol intoxication, as a consequence of alcohol metabolism, free radicals are generated and these processes are the most intensified in the liver (Lieber 1997). Moreover, the depletion in the liver antioxidant defense system, which is presented in this paper, was also observed earlier (Nordmann 1994; Scott et al. 2000). These changes result in enhanced reactions of free radicals with cell components mainly with phospholipid polyunsaturated fatty acids. In such a situation, especially important seems to be the decrease in the level/activity of antioxidants responsible for lipid protection observed in this paper. The main enzyme responsible for decomposition of peroxides including lipid peroxides is glutathione peroxidase. Diminution of GSH-Px activity is conductive to enhance lipid peroxide concentration and occurring chain reactions of lipid peroxidation. GSH-Px activity is manifested in the presence of reduced glutathione that is a cofactor of this enzyme (Oh et al. 1998). Therefore, the observed decrease in GSH level reduces the possibility of action of glutathione peroxidase and leads to an increase in the lipid peroxide level. Despite an increase in the activity of glutathione reductase, which is responsible for regeneration of reduced glutathione, chronic ethanol consumption causes the decrease in glucoso-6-phosphate dehydrogenase–the enzyme responsible for supplying NADPH needed for GSH regeneration (Oh et al. 1998), what caused that reduction of disulphide is impossible. Moreover, the glutathione peroxidase molecule contains selen in the active center. The oxidation of the cysteine SH-group adjacent to a selenol group or its modification by lipid peroxidation product the 4-hydroxynonenal may additionally affect the activity of glutathione peroxidase (Bosch-Morell et al. 1999). The decrease in the activity of antioxidant enzymes including glutathione peroxidase may be also caused by other factors during ethanol intoxication. It may be due to inhibition of protein molecule biosynthesis, which was earlier observed in ethanol intoxication (Bengston et al. 1984). Additionally, glutathione peroxidase and other antioxidative enzymes may be inactivated by the ethanol metabolite 1-hydoxyethyl radical (Puntarulo et al. 1999).

As a consequence of a decrease in antioxidant abilities observed during ethanol intoxication, membrane phospholipids are exposed to enhanced action of free radicals. The lipid peroxidation process is additionally intensified by a decrease in the level of vitamins, especially vitamin E, which acts as an efficient antioxidant in the lipid phase of biomembrane (Bradford et al. 2003). In the first step of this process, conjugated dienes and LOOH are generated and their levels are enhanced, as has been shown in this paper. It was proved that lipid hydroperoxides are decomposed in the transition metal ions' presence until carbonyl compounds are generated such as malondialdehyde or hydroxynonenal (Minotti 1993;Esterbauer et al. 1991), whose levels are enhanced during ethanol intoxication observed in this paper. These compounds are electrophilic and may form adducts with nucleophilic sulfhydryl, primary amino, and histydyl groups of proteins, which cause changes in protein structure and function (e.g., 4-HNE inhibits aldehyde dehydrogenase-mediated oxidation of ethanol to acetaldehyde) (Mitchell and Petersen 1987). Aldehydes generated during lipid peroxidation also form couplings with GSH, the main cellular nonenzymatic antioxidant, leading to a significant decrease in the cellular concentration of GSH, which may result in the cytotoxicity of oxidative stress. Moreover, these compounds yield a number of adducts with DNA (Chung et al. 1996, 2000; Marnett 1999). The compounds mostly react with DNA showing both genotoxic and mutagenic action; among them, 4-hydroxynonenal is most genotoxic while MDA is most mutagenic (Chung et al. 2000; Marnett 1999; Esterbauer et al. 1990). As a result of intensified lipid peroxidation in most of the examined tissues, resulting from ethanol metabolism, compounds revealing antioxidative properties are looked for, particularly those that occur in natural food products. Therefore, tea, being a very popular drink and containing big amounts of polyphenols characterized by strong antioxidative properties, has brought fresh hope to many research works in this field. Until recently most of the studies have dealt with antioxidative properties of green tea (Guo et al. 1996; Vayalil et al. 2003; Łuczaj et al. 2004; Ostrowska et al. 2004). However, independently of green tea, drinking black tea seems to be useful because of the protective effect of its components, which possess antioxidative properties (Łuczaj and Skrzydlewska 2005; Yoshino et al. 1994). Black tea components with proved antioxidant abilities are catechins (Rice-Evans et al. 1996). However, during the past years, it has been shown that the multimeric polyphenols of black tea (teaflavins and thearubigins formed during fermentation of tea leaves) possess even stronger antioxidant abilities than their precursors, catechins (Leung et al. 2001). Black tea polyphenols are absorbed from the gastrointestinal tract and are distributed into different tissues (Mulder et al. 2001; Warden et al. 2001). In the present study the level of black tea polyphenols (catechins as well as theaflavins) has been detected in the liver. It has been shown that teaflavins inhibit the activity of xanthine oxidase, the enzyme that generates the main and first formed of all radicals–the superoxide anion (Lin et al. 2000). However, if free radicals are generated, teaflavins are able to scavenge superoxide anion, singlet oxygen, and hydroxyl radical. These polyphenols react with these free radicals 10 times faster than the strongest antioxidant of all the catechins, EGCG (Jovanovic et al. 1997; Lin et al. 2000). In such a way black tea components may prevent free radical generation also enhanced during ethanol intoxication.

Moreover, catechins as well as teaflavins, as a result of their chelating effects, may diminish pro-oxidative action of transition metal ions, whose amount is increased during ethanol intoxication (Frei and Higdon 2003). Teaflavins also cause a decrease in iron absorption from the digestive tract (Hurrell et al. 1999). The reduced level of iron ions is unfavorable for free radical generation as well as for decomposition of lipid hydroperoxides, so the levels of lipid peroxidation products, the first (conjugated dienes and lipid hydroperoxides) and the final (MDA and HNE) ones, are decreased as the present study has revealed. Moreover, it can be suggested that black tea changes ethanol metabolism. However, it is only known that catechins, a minor fraction of black tea components, can inhibit the activity of cytochrome P450 2E1 (Goodin and Rosengren 2003), which participates in ethanol metabolism, especially in chronic intoxication. Consequently, the above effects reduce the generation of free radicals and decrease the possibility of their reaction with integral cell components.

In conclusion, black tea protects lipids of various rat tissues, especially the plasma, liver, stomach, and spleen, against ethanol-induced oxidative modifications and, consequently, prevents against changes in their biological functions. Considering that the metabolism of ethanol and tea polyphenols is the same in rats as in humans, the results obtained in the present study suggest that black tea may also protect human organs against the consequences of oxidative stress caused, for example, by ethanol intoxication.

## ABBREVIATIONS

BHT: butylated hydroxytoluene
EGCG: epigallocatechin gallate
NAD: nicotinamide adenine dinucleotide
NADH: reduced form of nicotinamide adenine dinucleotide
TF_1_: theaflavin
TF_2_: theaflavin gallate
TF_3_: theaflavin digallate
